# MP-Lasso chart: a multi-level polar chart for visualizing group Lasso analysis of genomic data

**DOI:** 10.5808/gi.22075

**Published:** 2022-12-30

**Authors:** Min Song, Minhyuk Lee, Taesung Park, Mira Park

**Affiliations:** 1 Department of Statistics, Korea University, Seoul 02841, Korea; 2 Department of Statistics, Seoul National University, Seoul 08826, Korea; 3 Department of Preventive Medicine, Eulji University, Daejeon 34824, Korea

**Keywords:** group Lasso, group structure, multi-level polar chart, sparsity, variable selection, visualization

## Abstract

Penalized regression has been widely used in genome-wide association studies for joint analyses to find genetic associations. Among penalized regression models, the least absolute shrinkage and selection operator (Lasso) method effectively removes some coefficients from the model by shrinking them to zero. To handle group structures, such as genes and pathways, several modified Lasso penalties have been proposed, including group Lasso and sparse group Lasso. Group Lasso ensures sparsity at the level of pre-defined groups, eliminating unimportant groups. Sparse group Lasso performs group selection as in group Lasso, but also performs individual selection as in Lasso. While these sparse methods are useful in high-dimensional genetic studies, interpreting the results with many groups and coefficients is not straightforward. Lasso's results are often expressed as trace plots of regression coefficients. However, few studies have explored the systematic visualization of group information. In this study, we propose a multi-level polar Lasso (MP-Lasso) chart, which can effectively represent the results from group Lasso and sparse group Lasso analyses. An R package to draw MP-Lasso charts was developed. Through a real-world genetic data application, we demonstrated that our MP-Lasso chart package effectively visualizes the results of Lasso, group Lasso, and sparse group Lasso.

## Introduction

In the analysis of high-dimensional genomics data, the least absolute shrinkage and selection operator (Lasso) method and its variants have been widely used to perform regression and model selection [1-3]. Typical Lasso performs variable selection at the individual gene level. However, genomic data can have a group structure, as in gene-expression data with pathways or in single-nucleotide polymorphism (SNP) data with genetic regions including multiple SNPs from genome-wide association studies. Better predictions can be expected if the group structure of the genes is considered. Several variants of Lasso have been developed to address the group structure [4-7]. Group Lasso ensures sparsity at the level of pre-defined groups and eliminates unimportant groups [4]. Moreover, sparse group Lasso combines Lasso and group Lasso to enable group selection as well as individual selection [5].

Visualization is used to effectively summarize the results of high-dimensional data analysis. The results of Lasso are often expressed as a tracking plot of regression coefficients. However, few studies have explored the systematic visualization of group information. In this study, we propose a multi-level polar chart for visualizing group Lasso analysis (the MP-Lasso chart). The MP-Lasso chart is an improved version of the MP chart, which was originally developed for integrating results from multi-omics data analyses [8]. An MP-Lasso chart shows at a glance the variables selected for each variable group and their coefficient estimates produced by group-structured penalized methods. We developed a program for creating MP-Lasso charts called “MP-Lasso” using R (https://github.com/statpark/MP-Lasso).

## Methods

### Penalized regression model

Consider a general linear model. We have *n* observations, and the data consist of *n*×1 including the response variable *y* and a *n*×*p* matrix *X* of predictors. Assume that *y* and X have been centered. The objective function of Lasso is


$$\underset{\beta }{\min}\;\;\left||y-X\beta \right|{|}_{2}^{2}\;+\lambda {\left|\left|\beta \right|\right|}_{1},$$

where *λ* is a tuning parameter, ||∙||_1_ stands for the vector *l_1_*-norm and ||∙||_2_ stands for the vector *l_2_*-norm. This penalty shrinks any coefficients contributing to the minimization problem to 0 [1].

Suppose that the predictors consist of *G* groups. Let *X_g_* be a matrix for the predictors of the g^th^ group with the corresponding coefficient vector *β_g_*. The objective function of group Lasso is


$$\underset{\beta }{\min}(||y-\sum_{g=1}^{G}X_{g}\beta_{g}|{|}_{2}^{2}+\lambda \sum_{g=1}^{G}\sqrt{p_{g}}\;|\left|\beta_{g}\right|{|}_{2}),$$

where $\sqrt{p_{g}}$ is the corresponding weight considering group size and ||∙||_2_ is the Euclidean norm [4,9]. Group Lasso shrinks all *β* values in irrelevant groups to 0. When *λ*=0, this criterion is equivalent to Lasso.

Sparse group Lasso uses a more general penalty to generate sparsity at both the group and individual feature levels, allowing the selection of groups and within-group variables. The objective function of sparse group Lasso is given by


$$\underset{\beta }{\min}\;\;(|\left|y-\sum_{g=1}^{G}X_{g}\beta_{g}|{|}_{2}^{2}+\left(1-\alpha \right)\lambda \sum_{g=1}^{G}\sqrt{p_{g}}|\left|\beta_{g}\right|{|}_{2}+\alpha \lambda \right|\left|\beta \right|{|}_{1}),$$

where *α*∈[0,1] and *β*=(*β*_1_,…,*β_G_*) [5,9]. *α* is a convex combination of Lasso and group Lasso. This criterion is equivalent to group Lasso if *α*=0, and to Lasso if *α*=1.

### MP-Lasso charts

An MP-Lasso chart consists of an outer level and an inner level. The outer level shows the overall impact of each group, and the inner level represents the impact of each variable within a group. For the outer level, the circle is divided into as many sectors as the number of groups. The segments in the chart are sorted by the maximum value of the coefficients in each group. A group with a rank of 1 starts at 0°. The radius of each sector is set to be proportional to the maximum value of the coefficients. When sorting or determining the radius, the maximum value may be replaced by the average. The number of variables in each group can be distinguished by the color of each segment.

For the inner level, points representing the variables in each group are plotted in each segment. The location of variables within each group is scaled by dividing the coefficient by the radius of the sector to represent the relative size. Different symbols are used according to the sign of the coefficient, and the number of variables belonging to each group is represented by the color spectrum.

**Fig. 1.**
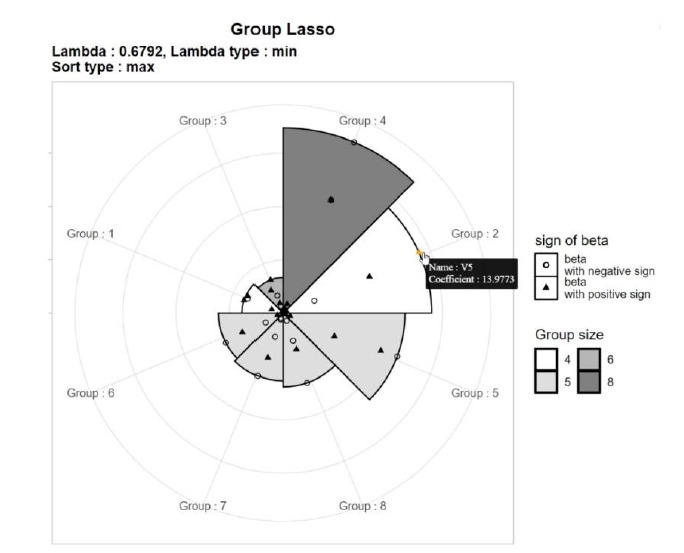
Example of multi-level polar least absolute shrinkage and selection operator (Lasso) chart using group Lasso analysis.

Each point is jittered slightly to avoid overlapping. Scatter plots are depicted in an interactive manner; moving the cursor on a point shows information about the variable. Fig. 1 shows an example of an MP-Lasso chart using results from group Lasso analysis with eight groups. Group 4 has the variables with the largest coefficients, followed by group 2, and so on. Group 2 contains four SNPs, among which the SNP with the largest regression coefficient is V5, with a regression coefficient of 13.9773.

## Results

### Implementation

We developed an R package to draw MP-Lasso charts. The program is available online (https://github.com/statpark/MP-Lasso). The MP-Lasso chart program requires two inputs: a cross-validated (CV) object and a group vector. A CV object can be obtained from the output objects of R packages, such as glmnet [10], gglasso [11], and SGL [12]. Cv.glmnet in glmnet, cv.ggLasso in ggLasso, and cvSGL in SGL perform Lasso, group Lasso, and sparse group Lasso analyses, respectively. The output objects contain regression coefficients with respect to sequential and optimal *λ* values that minimize CV error. The group vector represents the group structure of variables. Group names should be in character type or integer type. The group vector should be identical to the one used when the CV object is created. MP-Lasso chart supports three methods: Lasso, group Lasso, and sparse group Lasso. Table 1 summarizes the functions of the developed package for MP-Lasso charts and related packages to obtain input data.

**Fig. 2.**
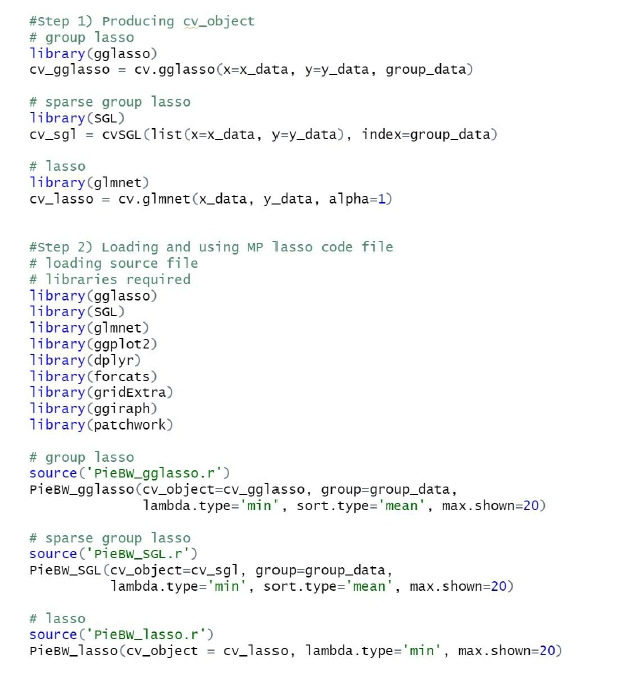
Example R code for creating cross-validated object.

Fig. 2 shows an example of code to obtain a CV object from the data. The group Lasso and sparse group Lasso methods require three inputs: predictor variables (x_data), dependent variables (y_data) and group vector data (group_data). The user can choose the number of folds k. It should be noted that the cvSGL function for sparse group Lasso takes input in list type only. For the Lasso model, *α* is set to 1 in the cv.glmnet function.

**Fig. 3.**
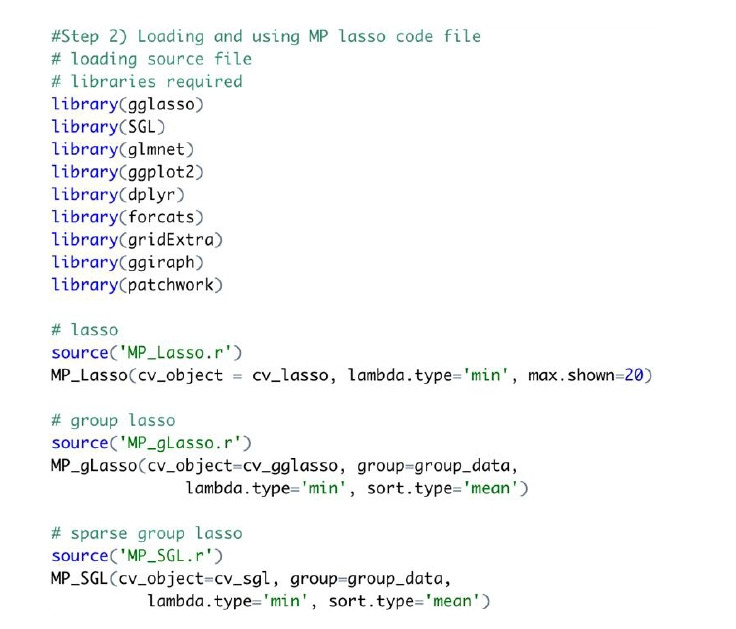
Example R code for multi-level polar least absolute shrinkage and selection operator chart.

An example of code for an MP-Lasso chart is shown in Fig. 3. The required libraries should be loaded before using the MP-Lasso R code. The function is required to match the Lasso method that creates the CV object. The same group vector used to create a CV object is also taken as input.

MP-Lasso chart has three options to determine the details of a plot. The lambda.type option decides which *λ* to use for each method, and it can take two values (“min” and “1se”), with “min” as default. The “min” option chooses the *λ* value that minimizes the CV loss. The “1se” chooses the largest *λ* with a CV error not 1 standard error further from the minimum CV loss. The “1se” option chooses fewer variables. The sort.type option determines which numeric feature represents the coefficients of variables in each group. Two choices are available for the sort.type option, “max” and “mean.” The “max” option uses the maximum absolute coefficient in each group as the feature of the group, while the “mean” option uses the mean of the absolute coefficients in each group. The last option is the max.shown option. When the number of chosen group is large, the chart can be too crowded with segments, making the chart difficult to interpret. By choosing max.shown, the user can decide the maximum number of segments shown on the chart. In the resulting MP-Lasso chart, interactive features are used. Moving the cursor over a point in the inner level displays information about that variable. For Lasso, moving the mouse over the sector area displays the corresponding information.

### Real data analysis

To illustrate the proposed MP-Lasso charts, we used T-cell and B-cell acute lymphocytic leukemia (ALL) data from the Ritz Laboratory [13]. The data consisted of microarray assays for 11,683 genes with 8,776 groups from 128 individuals with B-cell or T-cell ALL (https://bioconductor.org/packages/release/data/experiment/html/ALL.html). We conducted Lasso, group Lasso, and sparse group Lasso analysis for a binary phenotype using the glmnet, gglasso, and SGL packages. Each method is depicted using the *λ* value that yields the minimum 10-fold CV loss, and each group is represented by maximum absolute coefficients. For Lasso analysis, we set *max.shown*=30 for better representation. A summary of the results is presented in Table 2.

**Fig. 4.**
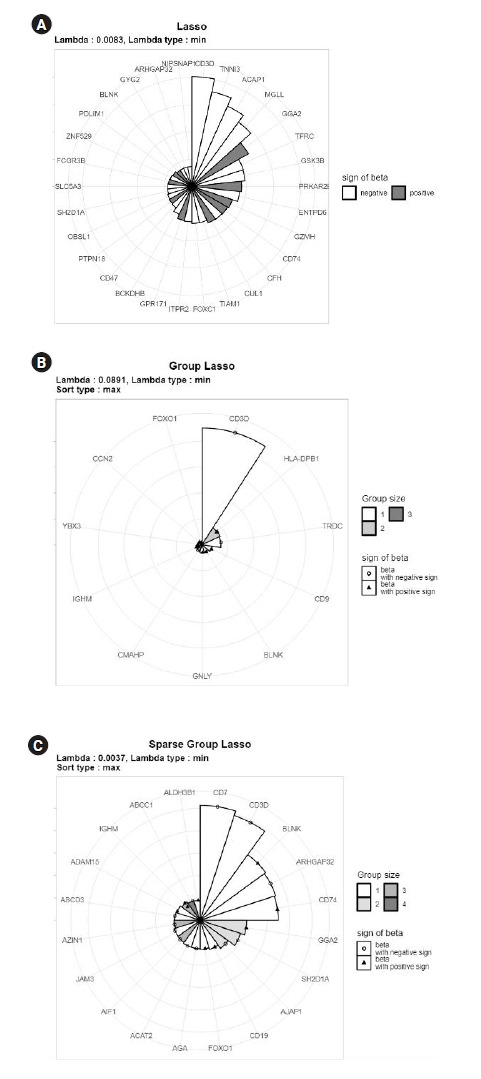
Multi-level polar least absolute shrinkage and selection operator (Lasso) chart for Lasso (A), group Lasso (C), and sparse group Lasso analysis of acute lymphocytic leukemia data.

Fig. 4 shows the resulting MP-Lasso chart sorted by maximum coefficients. Without group information, the Lasso analysis in Fig. 4A shows variables with the largest coefficients. The variables in the CD3D, TNNI3, and ACAP1 groups had the largest coefficients, in descending order. For group Lasso in Fig. 4B, the CD3D group had much larger maximum coefficients than the other groups and the next two groups (HLA-DPB1 and TRDC) had similar maximum coefficients to each other. Two variables in the HLA-DPB1 group had very similar coefficient values, which can be read from the position of points in the HLA-DPB1 sector. Fig. 4C shows the results from sparse group Lasso. Unlike in group Lasso, no single group dominated, and several significant groups remained. It can also be read that the first two groups had similar maximum coefficients. In this example, each group contained a small number of features. However, even if there are many variables in the groups, the color spectra are automatically adjusted so that the number of groups can be distinguished. Therefore, the user can recognize that a brighter segment indicates a smaller group, while a darker segment corresponds to a larger group. In conclusion, an MP-Lasso chart illustrates the different choices of groups and variables made by each model in detail.

## Discussion

We proposed a simple and efficient graph called an MP-Lasso chart for visualizing results from a group-penalized model. We also developed a corresponding R package. An MP-Lasso chart provides a clear representation of each group’s information and the relative importance of each variable within a group. Using our package, one can identify important groups and variables at a glance without having to check tables containing thousands of coefficients. It also facilitates model interpretation and comparisons of multiple models.

## Figures and Tables

**Table 1.** R packages and functions for MP-Lasso charts

Method	MP-Lasso chart function	Related package	
		Package	CV object function
Lasso	MP_Lasso()	glmnet	cv.glmnet()
Group Lasso	MP_gLasso()	ggLasso	cv.ggLasso()
Sparse group Lasso	MP_SGL()	SGL	cvSGL()

MP-Lasso, multi-level polar least absolute shrinkage and selection operator; CV, cross-validated.

**Table 2.** Top three groups with the highest maximum absolute coefficients

Method	Ranking	No. of variables in group	Group	Maximum absolute coefficients
Lasso	1	1	CD3D	0.121
	2	1	TNNI3	0.107
	3	1	ACAP1	0.097
Group Lasso	1	1	CD3D	–0.225
	2	2	HLA-DPB1	0.038
	3	1	TRDC	0.037
Sparse group Lasso	1	1	CD7	–1.280
	2	1	CD3D	–1.230
	3	1	BLNK	0.905

ALL, acute lymphocytic leukemia; Lasso, least absolute shrinkage and selection operator.
